# Chronotype, cognitive outcomes, and neural dynamics: recent evidence and potential mechanisms with implications for perioperative period

**DOI:** 10.3389/fnins.2025.1649396

**Published:** 2025-10-02

**Authors:** Ying Liu, Qijing Liu, Boxiong Gao, Qian Fu, Fang Li, Yuhu Ma, Yatao Liu

**Affiliations:** ^1^The First School of Clinical Medicine, Lanzhou University, Lanzhou, Gansu, China; ^2^Department of Anesthesiology and Operation, The First Hospital of Lanzhou University, Lanzhou, Gansu, China

**Keywords:** circadian rhythm, chronotype, melatonin, cognitive function, POCD

## Abstract

Circadian rhythm plays a fundamental role in regulating biological functions, including sleep–wake preferences, body temperature, hormone secretion, food intake, cognitive function and physical performance. The sleep chronotype, as part of the circadian rhythm, usually refers to an individual’s subjective preference for their own sleep–wake cycle. Because of the differences in brain microstructure and resting-state connections between different sleep chronotype, it may lead to differences in individual cognitive function. Concurrently, the pathophysiological mechanisms underlying the association between perioperative circadian misalignment and postoperative cognitive dysfunction (POCD), as well as targeted therapeutic strategies, have garnered increasing attention in recent research. Chronotype exerts regulatory effects on cognitive function via circadian rhythm modulation, neuroinflammatory cascades, and metabolic homeostasis. Perioperative alterations in sleep architecture—including diminished slow-wave sleep (SWS) and circadian desynchronization—may potentiate cognitive deficits and exacerbating neuroinflammation-mediated neuronal apoptosis. This review mainly focuses on the relationship between sleep chronotype and cognitive function as well as perioperative sleep chronotype changes, providing the latest evidence of relevant studies of domestic and foreign. In addition, different sleep patterns and postoperative cognitive dysfunction are prospected, which provides a new direction for exploring the different mechanisms of postoperative cognitive dysfunction in the future.

## 1 Introduction

Human nature has temporal components. Rhythm can be found at different organizational levels, in fact, almost all physical and mental functions change periodically. One of the most well-known is circadian rhythm, circadian rhythm is a 24 h cycle of life activities change rule which is synchronized by environmental signals (zeitgebers). Sleep–wake cycle is co-regulated by circadian oscillator and steady-state oscillator, when awake time reaches a certain length of time, sleep homeostasis begins to occupy the dominant position, with the accumulation of sleep homeostasis time, it is transformed into a circadian rhythm dominated, the two restrict each other, jointly determine the sleep demand (Borbély et al., 2016).

Human circadian rhythm is represented by a inherently complex phenotype derived from multiple underlying genetic factors that define the chronotype. Recent studies have found that individual’s cognitive task performance is significantly related to their sleep–wake preference time, and the brain’s neuroplasticity and excitability are higher when the learning task is completed at the “best time” mapped by the individual’s sleep chronotype (Salehinejad et al., 2021). Besides, researchers have found that there are age and population differences in the relationship between sleep chronotype and cognitive function (Ujma and Scherrer, 2021; Wang et al., 2022).

However, although some observational studies have shown that cognitive function is related to chronotype, few reviewers have systematically summarized the existing research results on chronotype and cognition. Especially in the aging society, with the continuous development of science and technology, the volume of surgery is increasing. It is particularly important to pay attention to the cognitive function of perioperative elderly patients.

This narrative review aims to summarize recent studies on chronotype and examine its relationship with cognitive function. The structure is as follows. We first described the definition, relevant evaluation methods and influencing factors of sleep chronotype. Then, we reviewed studies on sleep chronotype and cognitive function to clarify the potential mechanism. In the third section, we discussed the change of sleep midpoint in perioperative patients, which led to thinking about whether the chronotype changes in perioperative period. Finally, we concluded with a summary of the current state of research, identifying gaps and providing suggestions regarding future research.

## 2 Chronotype

### 2.1 Overview

#### 2.1.1 Conception

Sleep chronotype, also as known as circadian preference, refers to individuals’ subjective preference for their own sleep–wake cycle, often showing significant individual variation, and is part of circadian rhythm (Adan et al., 2012). Defined as individual differences of the preferred timing of the sleep–wake cycle (Zavada et al., 2005), reflect the properties of an individual’s circadian phase (Levandovski et al., 2013), which reveals how active an individual is throughout the day in terms of body state, hormone levels, core body temperature, cognition function, diet, and sleep quality.

#### 2.1.2 Classification

Recent studies commonly divide chronotype into three (or five) types: morning chronotype (moderate morning chronotype and definite morning chronotype), evening chronotype (moderate evening chronotype and definite evening chronotype), and intermediate chronotype (Horne and Ostberg, 1976).

(1) Morning type: Also known as early chronotype or larks. Morning type individuals are more active in the morning, they usually prefer to go to bed early and get up early. In the early part of the day after waking up, they achieve peak physical and mental performance.

(2) Evening type: Also called late chronotype or owls, prefer to be active in the evening and sleep and wake up late. Contrary to the morning type, evening chronotype individuals have the best mental and physical performance before sleeping.

(3) Intermediate type: Also regarded as neutral or neither type. This type of crowd has no preference for morning or evening.

### 2.2 Assessment methods

#### 2.2.1 Subjective evaluation methods

Chronotype can be evaluated using self-reported questionnaires, the following scales are the most widely used: Morningness-Eveningness Questionnaire (MEQ) (Horne and Ostberg, 1976), Composite Scale of Morningness (CSM) (Smith et al., 1989), Munich Chronotype Questionnaire (MCTQ) (Roenneberg et al., 2003), and recently improved scale—Morningness-Eveningness-stability Scale (MESSi).

#### 2.2.2 Objective evaluation methods

Other relative objective physiological indexes can evaluate chronotype, including dim light melatonin onset (DLMO), the serum cortisol and core body temperature. In addition, it can also use polysomnography (PSG) and actigraphy to record sleep chronotype.

#### 2.2.3 Comparation

Due to the high cost and complex operation of objective methods, their application in large-scale research is limited. In contrast, using subjective scales to assess sleep chronotype is easier and more feasible, so they can be used in large-scale clinical researches and epidemiological investigations. One of the most widely used questionnaires is MEQ and MEQ-5, which uses a statistical modal to extract five items of MEQ. It has been proved by researches that MEQ-5 has good psychometric characteristics (Danielsson et al., 2019). At the same time, the chronotype obtained by the objective measurement of sleep–wake cycle using the actigraphy are basically consistent with the results of the self-assessment questionnaires (Thun et al., 2012).

### 2.3 Influencing factors

**Figure 1:**
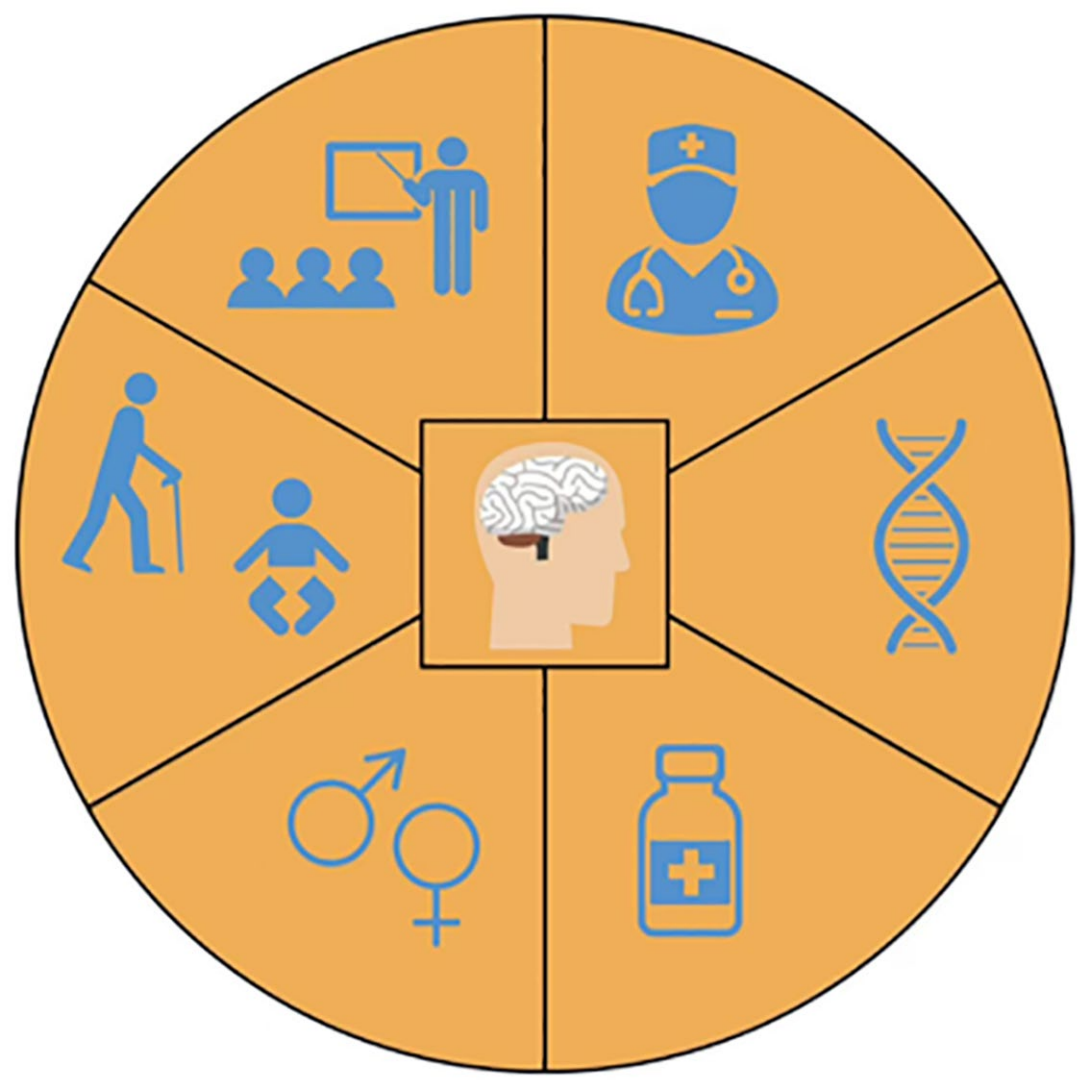
Factors influencing the sleep–wake cycle and sleep chronotype.

Sleep chronotype is regulated by many factors such as physiology, heredity, behavior (Figure 1).

#### 2.3.1 Gender and age

Gender has been proven to correlated with chronotype. In adults, morningness predominates among females (Vink et al., 2001), and high levels of testosterone seem to lead to a stronger tendency toward eveningness among males (Randler et al., 2012), although the difference disappears in menopause or elderly age. In addition, the changes in the propensity toward preferring the morning and the evening that occur with advancing age are perhaps due to changes in hormone secretion (Hagenauer et al., 2011).

#### 2.3.2 Melatonin and other hormones

Melatonin, also known as the pineal hormone, is an indole-like neuroendocrine hormone secreted by the pineal gland (Guo et al., 2023). Melatonin has widespread effects on the body and acts as a neuroendocrine transducer and circadian signal, is used as one of the biological markers of morningness-eveningness. Morning types have an advance in the melatonin secretion rhythm phase compared to evening types (Allada and Bass, 2021): compared with the evening type, the onset, acrophase, and offset of the melatonin profiles occurs approximately 3 h earlier in morning type, with no differences in amplitude. Because the evening type has higher melatonin levels at 9 a.m. than morning type, previous studies have shown that serum melatonin concentrations measured at 9 a.m. may help distinguish different sleep chronotype. At noon, the serum melatonin level typically does not show any differences between morning types and evening types anymore (Morera-Fumero et al., 2013).

Apart from melatonin, which clearly reaches its peak plasma concentration between 2 and 4 a.m., the nocturnal oscillations of other hormones also play a significant role in the generation and regulation of sleep patterns. Cortisol shows a characteristic rhythm of a peak in the morning and a trough at night. Among sex hormones, the level of testosterone is similar to that of cortisol, being the highest in the early morning and the lowest at night. Growth hormone is mainly secreted in a pulsatile manner during slow-wave sleep and is most vigorously secreted in the first sleep cycle at night (Begemann et al., 2025). These hormones, which have distinct circadian rhythms in production and secretion, are the core regulatory factors for maintaining normal circadian rhythms and sleep chronotype, and have a profound impact on various pathological and physiological processes in the human body. Although more extensive research is still needed to describe the connection between circadian clock and skin wound healing, an existing study have shown that in female Siberian hamsters, wounds incurred at night take longer to recover by 50% than wounds incurred at day (Cable et al., 2017).

#### 2.3.3 Surgery and anesthesia

Suprachiasmatic nucleus neurons contain *N*-methyl-d-aspartic acid (NMDA) and γ-aminobutyric acid (GABA) receptors, and the activation of these receptors affects the expression of clock genes and the synchronization between the internal clock and the exogenous clock. Most of the drugs of anesthesia are NMDA receptors’ antagonists and GABA receptors’ agonists. Clinical observational researches have found that anesthesia and surgery can cause delays in the endogenous rhythm of plasma melatonin levels and melatonin metabolite excretion (Gögenur et al., 2007). In addition, glucocorticoids, which are used in surgery, could mimic endogenous cortisol and have a strong effect on the molecular clock (Oster et al., 2017). Numerous studies have assessed effects of anesthesia and surgery on circadian rhythm; however, research on how does anesthesia/surgery affect perioperative chronotype is scarce.

#### 2.3.4 Clock genes

Clock genes, including core regulators CLOCK, BMAL1, and feedback components Period (PER) and Cryptochrome (CRY), orchestrate circadian rhythms through a transcription-translation feedback loop (TTFL). The CLOCK/BMAL1 heterodimer activates downstream targets (e.g., Rev-reba, Dbp) via E-box binding, while PER/CRY proteins inhibit CLOCK/BMAL1 activity, establishing 24-h rhythmicity. Genetic studies reveal that the CLOCK genes’ polymorphism is associated with delayed sleep phase (Jones et al., 2019). BMAL1 knockout models exhibit sleep fragmentation and reduced NREM sleep underscoring clock genes’ direct role in chronotype regulation (Qiu et al., 2019).

#### 2.3.5 Ion channels

Ion channels are not only the output pathways of the biological clock but also can feedback regulate the core clock mechanism. Data collected in the last decade, including different model organisms, support a central role for metal ions in regulating gene expression rhythms of core clock genes, both at the transcription and translation level (Stangherlin, 2023). Studies have shown that I_Ca(L)_ and fast delayed rectifier K^+^ currents [I_K(FDR)_] inhibition affect firing during the day, when their respective current magnitudes are larger, and I_BK_ inhibition affects firing at night, when its current magnitude is larger (Harvey et al., 2020). This ion current rhythm with diurnal periodic changes is also very likely to be the basis of sleep chronotype, but further research is still needed.

In addition to the aforementioned metal ion channels, another type of ion channel has been discovered to serve as a bridge between body temperature and the circadian rhythm—transient receptor potential (TRP) channels. This is a molecular mechanism whereby external light and temperature are able to communicate sleep and wakefulness through multiple clock genes and TRP channels located throughout the body including muscle cells, neuronal cells and peripheral nerves sending cues as to time of day (Woodard et al., 2024).

#### 2.3.6 Social jetlag

Social jetlag (SJL) is a proxy for circadian misalignment quantifying the discrepancy between social and biological time (Roenneberg et al., 2019). Chronic SJL induces circadian phase delay and amplitude dampening, disrupting sleep homeostasis and metabolic regulation. The interplay between SJL and clock genes exacerbates chronotype disruption. Genome-wide association studies (GWAS) demonstrate that carriers of the CRY1 rs2287161 risk allele exhibit aggravated melatonin rhythm dysregulation under chronic SJL, with dose-dependent correlations to subjective sleep quality deterioration.

### 2.4 Chronotype and disorders

Sleep and awakening are important prerequisites for cognitive efficiency, and the decline of cognitive efficiency can seriously affect individual emotion and performance, leading to the occurrence of related diseases.

#### 2.4.1 Sleep disorders

Differences between sleep chronotype can cause circadian rhythm sleep–wake disorders (CRSWDs), advanced sleep–wake phase disorders (ASWPD) and delayed sleep–wake phase disorders (DSWPD) all three of these clinical diagnoses (Facer-Childs et al., 2019). Patients with DSWPD tend to shorten the length of their sleep because of social constraints (such as work/school regulations), leading to the accumulation of sleep debt, which leads to excessive daytime sleepiness and impaired cognitive function, many of which are similar to those of evening type individuals.

#### 2.4.2 Depressive disorders

Previous studies have shown that different sleep chronotypes in univariate analysis have significant effects on the occurrence of depressive symptoms and anxiety, and after controlling other influencing factors, further multivariate logistic regression analysis shows that evening types is one of risk factors for both depression and anxiety. This may be due to the differences in activities and functional connections in brain regions involved in emotional processing. Morphometric studies of the brain have identified associations of sleep chronotype with various subcortical structures and hippocampal subfields. The evening chronotype group exhibited notable reductions in the right caudate and the left strata radiatum/lacunosum/moleculare (SR-SL-SM). Additionally, the amplitude scale of the Chronotype Questionnaire (CHQ-AM) demonstrated significant positive and negative correlations with the volumes of the left thalamus and amygdala, respectively (Alhazmi, 2025). Moreover, evidence indicates that patients with major depressive disorder (MDD) exhibit dysregulated expression of clock genes in the dorsolateral prefrontal cortex, hippocampus, amygdala, nucleus accumbens, and cerebellum (Li et al., 2013). The amygdala is an important response organ in the limbic system of the brain, and its activation can be used as an indicator of the emotional intensity of an individual under stress (Frank et al., 2014).

With the increasing complexity of social relations and the increasing pressure of life, the incidence of depression disorders is rising day by day. Depressive disorders are characterized by significant and persistent depressive symptoms as the primary clinical feature, often accompanied by the rhythmical fluctuation of mood and high morbidity, heavy burden, and great social attention. Many researches have shown that evening-type individuals are significantly associated with depression, one of the recent meta analyses of 43 studies revealed a reliable association between a preference for night sleep patterns and depression symptoms (Norbury, 2021).

#### 2.4.3 Bipolar disorders

Bipolar disorders (BD), including BD-I and BD-II, are mood disorders. Previous studies have confirmed the association between chronotype and BD after measuring sleep chronotype using subjective methods (such as questionnaires) and objective methods (such as actigraphy) (Gershon et al., 2018; Kaufmann et al., 2018). However, other researches have found no significant correlation between chronotype and the two BD subtypes. In conclusion, sleep chronotype may be a risk factor for BD because of its more severe symptoms and more complications. Nevertheless, the current evidence is insufficient and the underlying mechanism is not clear. In the future, a large number of relevant studies are still needed to pay attention to the relationship between chronotype and BD.

## 3 Chronotype and cognitive function

### 3.1 Outline

As people get older, cognitive function declines each year. According to the World Health Organization’s 2022 blueprint for dementia research, an estimated 55.2 million individuals globally are affected. The prevalence among those over the age of 60 varies by region: with Southwest Asia reporting a prevalence of 2.9%, Europe at 6.5%, and other regions experiencing rates between 3.1 and 5.7%. In the United States, approximately one in nine individuals (10.8%) age 65 and older suffer from Alzheimer’s disease (AD), with an annual incidence of 1,275 new cases per 100,000 persons (Zhang et al., 2024). As an external characteristic of individual sleep rhythm preference, the age-related changes of chronotype usually begin to change after the age of 40–50 years (Taillard et al., 2021). At present, there are some researches on sleep chronotype and cognitive function, which suggest that chronotype is related to cognitive function.

### 3.2 Related researches

In recent years, it has been found that in traditional regression analysis, the evening chronotype is associated with better cognitive function in elderly individuals (Wang et al., 2022), specifically, in unadjusted model, participants with per hour later sleep midpoint had higher Mini-Mental State Examination (MMSE) and Delayed Word Recall Test (DWRT) scores, and this association attenuated but remained significant after adjusting for sex, age, education, occupation, BMI, self-reported health, alcohol use, smoking status, physical activity, cardiovascular disease history, diabetes history, depressive symptoms and sleep duration. In bidirectional two-sample Mendelian randomization (MR) analyses, through adding by providing causal effects of cumulative exposures across the life course, rather than the effects at a specific short time, suggesting that lifetime better cognitive function is associated with later chronotype. Secondly, a 2017 cross-sectional research conducted by the UK Biobank with a sample size of 477,529 found that evening sleep types performed better on all cognitive tasks except digital memory, while morning sleep types had worse cognitive function than intermediate sleep types (Kyle et al., 2017). As one manifestation of cognitive function, the relationship between executive functioning and chronotype was also demonstrated in a recent study with a sample size of 180. When executive function was assessed as a whole, evening chronotypes exhibited better performance in information processing speed compared to intermediate and morning types. Morning chronotypes were associated with difficulty sustaining attention, low verbal fluency, and poor executive control skills (Demirci et al., 2025). Whereas, a recent study that included 224,714 people who had a routine checkup showed that the risk of cognitive decline was significantly different by sleep hours and morningness-eveningness type (Ahn et al., 2024). Regardless of the sleep quality, 7–8 sleep hours had the lowest risk of cognitive decline, and the morningness type had a lower risk of cognitive decline than the intermediate type or eveningness type. However one limitation of this study is that chronotypes were classified according to wake up time and did not use subjective scales or genetic variants, which may have resulted in a bias in the results. The relationship between chronotype and cognitive function highlights the intricate effects of circadian regulation on neurobehavioral outcomes, yet its mechanisms and clinical implications remain debated. Future research should integrate multimodal data (e.g., dynamic EEG, metabolomics) and longitudinal cohorts to unravel the temporal sensitivity and individualized intervention thresholds of the chronotype-cognition axis.

### 3.3 Underlying mechanisms

Technological advances in human cognitive science have sparked new interest in the effects of “chronotype” and “circadian rhythms” on human brain physiology and cognition (Ly et al., 2016; Schmidt et al., 2007). Given that modern lifestyles are increasingly less dependent on 24-h circadian rhythms, a further understanding of how human brain and cognitive function are affected by the body clock and sleep chronotype has profound implications for public health, the work environment, academic performance, and the pathophysiology of related diseases (Takahashi et al., 2008; Scheiermann et al., 2013; Heyde et al., 2018).

#### 3.3.1 Neural basics of chronotype

With regard to brain structure, it is well known that brain regions are connected and innervated via white matter (WM) bundles, so the structure of WM is essential for coordinating brain function. In one study, after exploring the microstructures of WM through diffusion tensor imaging (DTI), it was found that the frontal lobe, temporal lobe, corpus callosum and other regions showed significant differences in the WM integrity structure of different chronotypes, as manifested in the following aspects: Compared with early chronotypes and intermediate chronotypes, evening chronotypes in the left anterior cingulate gyrus (ACC) at the bottom of the WM showed a significantly lower of the fractional anisotropy (FA) and lower fiber count (FC) values in WM below the right frontal lobe (Rosenberg et al., 2014). By the way, WM microstructure underlying the frontal lobes has been associated with disturbances in motor movements and cognitive functions, e.g., attention (Kiernan and Hudson, 1994).

Brain lateralization denotes the functional asymmetry between the cerebral hemispheres in cognitive, sensory, and motor processing. Research on this topic dates back to the mid-19th century (Güntürkün et al., 2020). Recent evidence reveals marked hemispheric asymmetries in individuals with different sleep chronotype. Employing voxel-based morphometry (VBM) and vertex-wise cortical thickness (CTh) analysis, Rosenberg et al. noted that early chronotypes showed significantly lower gray matter volumes in the right lingual gyrus, occipital fusiform gyrus and the occipital pole as compared to intermediate chronotype. Furthermore, lower gray matter volumes for early chronotypes in the left anterior insula, precuneus, inferior parietal cortex, and right pars triangularis than for late chronotypes (Rosenberg et al., 2018). Another study published in 2025 similarly demonstrated that a notable leftward hemispheric laterality of the subiculum was found in the early chronotype group compared to the late chronotype group (Alhazmi, 2025). These findings may reveal the basis for chronotype differences in brain function and shed how sleep chronotype affects brain lateralization.

Regarding the basis of brain imaging, both morning and evening chronotypes are significantly associated with functional connectivity of the default mode network and frontoparietal network (DMN-FPN), and the more early morning type individuals tend to have stronger functional connectivity (Hodkinson et al., 2014). The correlation between the morning chronotype and the functional connection of the left dorsolateral superior frontal-left inferior parietal marginal angular gyrus may mainly reflect the role of internal circadian rhythm. The negative correlation between evening chronotype and functional connection between left angular gyrus and right frontal middle gyrus may mainly express the components of homeostasis process.

#### 3.3.2 Possible underlying mechanisms of the connection between chronotype and cognitive function

The chronotype and the functional connection of brain’s resting state can be used to predict human’s cognitive performance (Facer-Childs et al., 2019). Salehinejad et al. (2021) conducted a successful trial using non-invasive brain stimulation (NIBS) and transcranial direct current stimulation (tDCS) to monitor people with different sleep chronotypes and found that motor learning and cognitive performance (working memory, and attention) along with their electrophysiological components are significantly enhanced at the circadian-preferred, compared to the non-preferred time. This outperformance is associated with enhanced cortical excitability (prominent cortical facilitation, diminished cortical inhibition), and long-term potentiation/depression-like (LTP/LTD) plasticity (Salehinejad et al., 2021). For cortical-related electrophysiological changes, at the circadian-preferred time, intracortical facilitation is enhanced predominantly by increased activity of glutamatergic synapses. Conversely, cortical inhibition is significantly pronounced at the circadian non-preferred time presumably through enhanced GABAergic activation. In the second place, for the regulation of neuroplasticity, this study found that LTP/LTD-like neuroplasticity also depends on glutamatergic and GABAergic systems and is driven by NMDA receptors. This is consistent with the evidence from primary motor cortex modals in humans and animals (Castañeda et al., 2004; Albus et al., 2005; Langel et al., 2018).

At the same time, another research has found that compared with the morning chronotype, the cognitive ability and emotional experience of the evening chronotype are more likely to be impaired. And then after the intervention based on light and sleep homeostasis pressure, the cognitive ability, emotional problems, sleep quality and sleep structure of the evening chronotype subjects were significantly improved. It may be that the prolonged sleep duration after the intervention causes the regulation of neurotrophic factors, improves the glucose metabolism in the brain, down-regulates the adenosine concentration, and then attenuates the negative effects of homeostasis sleep stress to improve cognitive function (Arnal et al., 2015).

## 4 The changes of chronotype during perioperative period

The midpoint of sleep is one of the main evaluation criteria of chronotype. Since the central regulator of chronotype, suprachiasmatic nucleus neurons, contains NMDA receptors and GABAergic, and most of the drugs used in general anesthesia are NMDA receptor antagonists and GABAergic agonists, anesthesia is likely to disrupt individual chronotype. In animal studies, it has been confirmed that many anesthetic drugs such as isoflurane, sevoflurane, propofol, ketamine, etc., can induce strong circadian phase shift, meaning that the midpoint of sleep is advanced or delayed (Orts-Sebastian et al., 2019).

**Figure 2:**
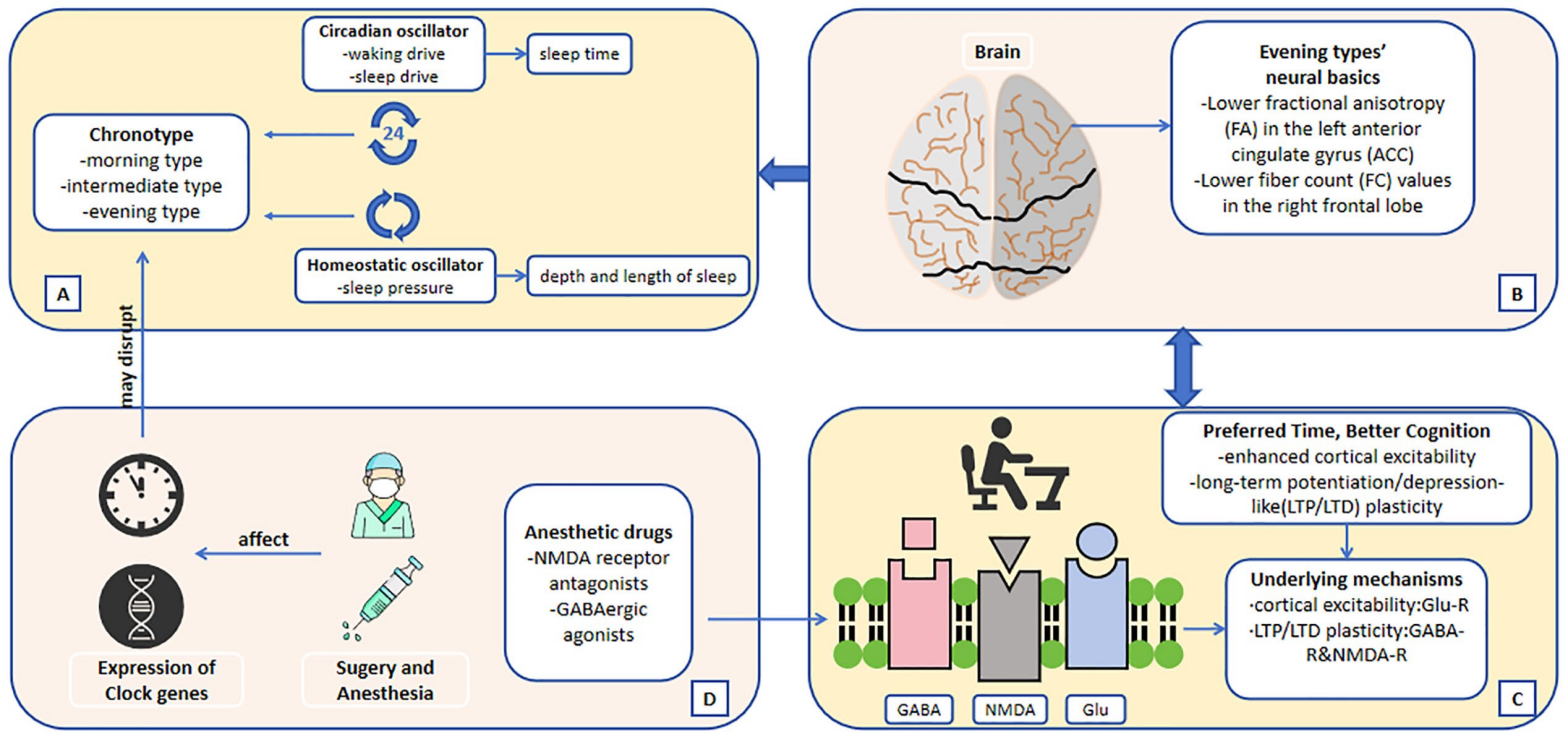
**(A)** The two oscillators regulate sleep–wake cycle and lead to chronotype. **(B)** Neural basics of different chronotypes. **(C)** Possible underlying mechanisms of the connection between chronotype and cognitive function. **(D)** The mechanisms by which surgery and anesthesia may affect sleep chronotype. The direction of the arrows indicates the possible causality.

In 2022, an observational study of 94 patients found that surgery/anesthesia could cause a shift in sleep time (van Zuylen et al., 2022). The later the patients’ midpoint of sleep before surgery, the more advance the midpoint of sleep after surgery or general anesthesia. On the contrary, if the patients’ preoperative chronotype is more inclined to the morning type, the advance of the midpoint of sleep duration after surgery or general anesthesia is small, or even delayed the situation. Potential mechanisms include time-dependent effects of propofol administration or perioperative stress on the suprachiasmatic nucleus. And just this year, the team behind this trial published a follow-up research on perioperative sleep changes (Meewisse et al., 2025). After expanding the sample size and considering the timing of surgery, the results were consistent with those of the previous study: a mean phase advance in midpoint of sleep of approximately 30–40 min on the night following surgery and an associated decline in sleep quality for all patients. Moreover, this follow-up research included chronotype in the baseline data and found that, patients with later chronotype experienced larger phase advances (the specific performance is a larger phase advance of sleep–wake rhythm). This result might be related to the more difficult adjustment of this type of patients to inpatient hospital schedules. In addition to exploring more non-cardiac operations, another clinical study on the effect of intravenous anesthesia on the circadian rhythm of patients undergoing cardiac closure mentioned that the sleep midpoint of patients 1 week after surgery was about 21 min earlier than that before surgery, and with the increase of the duration of anesthesia, the sleep midpoint of patients was more advanced and more close to the morning type. This result suggested that intravenous anesthesia may improve sleep habits by compensating sleep debt and advancing sleep chronotype (Gu et al., 2024). Apart from clinical studies, there is now a substantial body of evidence summarizing the shifting effect of general anesthesia on the circadian clock in a range of vertebrate and invertebrate species. The proposed mechanism of this effect is via anesthetic agents acting on the expression of core circadian clock genes (Figure 2) (Poulsen et al., 2018).

The sleep midpoint, serving as a critical metric for assessing circadian phase alignment, frequently represents a concrete manifestation of altered sleep patterns. Consequently, perioperative shifts in sleep midpoint serve as a significant marker of circadian rhythm disruption in surgical patients. This disruption manifests as diminished subjective sleep quality, reduced sleep efficiency, increased daytime fatigue (van Zuylen et al., 2022), and potentially prolonged postoperative recovery.

## 5 Prospective: the relationship between sleep chronotype and postoperative cognitive dysfunction

Postoperative Cognitive Dysfunction (POCD), a complex postoperative complication, has attracted more and more attention in recent years. Foreign studies have revealed that the incidence of POCD in patients with digestive system tumors can reach more than 30%, which may be related to the tumor patients’ age, tumor immune mechanism, and duration of surgery and anesthesia (Rundshagen, 2014; Evered et al., 2011; Xi et al., 2021). Symptoms usually appear in the first few days or mouths after surgery, and are mainly manifested as memory, abstract thinking and orientation disorders after surgery, accompanied by deceased social activity ability, which affect postoperative recovery, lead to related complications and even affect the middle and long term prognosis of patients (Niu et al., 2021). While some patients’ symptoms will resolve over time, even few patients’ cognitive dysfunction can last for years. Although a large number of clinical and basic studies have been conducted on the pathogenesis of POCD, the etiology, pathogenesis and risk factors of POCD have not been fully explained. Among them, the association between circadian rhythm and POCD is one of the recent research hot topics.

For the past few years, although some scholars have paid attention to the change of sleep chronotype in postoperative patients, it seems that few clinical researches have explored the connection between sleep chronotype and postoperative cognitive dysfunction. A systematic review published last year on the effects of timing for elective (non-cardiac) surgery on mortality, complications, and other relevant clinical outcomes noted that the role of patients’ chronotype in the context of optimal timing for surgery has not been explored, and that including patients’ chronotype in future studies may provide new insights into the effects of timing on surgical outcomes (Meewisse et al., 2024). On the other hand, most anesthetic drugs are NMDA receptor antagonists or GABAergic agonists, which share many similar pathways with the mechanism of sleep chronotype on cognitive function mentioned above. However, whether anesthetic drugs will affect postoperative cognitive function of patients by changing sleep chronotype has not been clearly determined.

Therefore, in the future, the relationship between different chronotype and POCD and its related mechanisms still need to be further explored in a large number of multi-center and large-sample clinical researches.

## References

[ref1] Adan A. Archer S. N. Hidalgo M. P. Di Milia L. Natale V. Randler C. (2012). Circadian typology: a comprehensive review. Chronobiol. Int. 29, 1153–1175. doi: 10.3109/07420528.2012.719971, PMID: 23004349

[ref2] Ahn E. K. Yoon K. Park J. E. (2024). Association between sleep hours and changes in cognitive function according to the morningness-eveningness type: a population-based study. J. Affect. Disord. 15, 112–119. doi: 10.1016/j.jad.2023.10.122 37865346

[ref3] Albus H. Vansteensel M. J. Michel S. Block G. D. Meijer J. H. (2005). A GABAergic mechanism is necessary for coupling dissociable ventral and dorsal regional oscillators within the circadian clock. Curr. Biol. 15, 886–893. doi: 10.1016/j.cub.2005.03.051, PMID: 15916945

[ref4] Alhazmi F. H. (2025). Exploring the correlation between sleep Chronotype and the volumes of subcortical structures and hippocampal subfields in young healthy population. Brain Sci. 15:295. doi: 10.3390/brainsci15030295, PMID: 40149816 PMC11940034

[ref5] Allada R. Bass J. (2021). Circadian mechanisms in medicine. N. Engl. J. Med. 384, 550–561. doi: 10.1056/NEJMra1802337, PMID: 33567194 PMC8108270

[ref6] Arnal P. J. Sauvet F. Leger D. van Beers P. Bayon V. Bougard C.. (2015). Benefits of sleep extension on sustained attention and sleep pressure before and during Total sleep deprivation and recovery. Sleep 38, 1935–1943. doi: 10.5665/sleep.5244 26194565 PMC4667385

[ref7] Begemann K. Rawashdeh O. Olejniczak I. Pilorz V. de Assis L. Osorio-Mendoza J.. (2025). Endocrine regulation of circadian rhythms. Npj biol timing. Sleep 2:10. doi: 10.1038/s44323-025-00024-6 PMC12912344 41775850

[ref8] Borbély A. A. Daan S. Wirz-Justice A. Deboer T. (2016). The two-process model of sleep regulation: a reappraisal. J. Sleep Res. 25, 131–143. doi: 10.1111/jsr.12371, PMID: 26762182

[ref9] Cable E. J. Onishi K. G. Prendergast B. J. (2017). Circadian rhythms accelerate wound healing in female Siberian hamsters. Physiol. Behav. 15, 165–174. doi: 10.1016/j.physbeh.2016.12.019 PMC5619253 27998755

[ref10] Castañeda T. R. de Prado B. M. Prieto D. Mora F. (2004). Circadian rhythms of dopamine, glutamate and GABA in the striatum and nucleus accumbens of the awake rat: modulation by light. J. Pineal Res. 36, 177–185. doi: 10.1046/j.1600-079X.2003.00114.x, PMID: 15009508

[ref11] Danielsson K. Sakarya A. Jansson-Fröjmark M. (2019). The reduced Morningness-Eveningness questionnaire: psychometric properties and related factors in a young Swedish population. Chronobiol. Int. 36, 530–540. doi: 10.1080/07420528.2018.1564322, PMID: 30614272

[ref12] Demirci H. Bilge Y. Şirin M. Sarıkaya A. F. İlhan S. (2025). Chronotype and cognition: comparison of executive functions, sleepiness, and fatigue according to circadian rhythm preference. J Turk Sleep Med. 12, 70–80. doi: 10.4274/jtsm.galenos.2024.26214

[ref13] Evered L. Scott D. A. Silbert B. Maruff P. (2011). Postoperative cognitive dysfunction is independent of type of surgery and anesthetic. Anesth. Analg. 112, 1179–1185. doi: 10.1213/ANE.0b013e318215217e, PMID: 21474666

[ref14] Facer-Childs E. R. Campos B. M. Middleton B. Skene D. J. Bagshaw A. P. (2019). Circadian phenotype impacts the brain’s resting-state functional connectivity, attentional performance, and sleepiness. Sleep 42:zsz033. doi: 10.1093/sleep/zsz033 30763951 PMC6519915

[ref15] Facer-Childs E. R. Middleton B. Skene D. J. Bagshaw A. P. (2019). Resetting the late timing of ‘night owls’ has a positive impact on mental health and performance. Sleep Med. 60, 236–247. doi: 10.1016/j.sleep.2019.05.001, PMID: 31202686

[ref16] Frank D. W. Dewitt M. Hudgens-Haney M. Schaeffer D. J. Ball B. H. Schwarz N. F.. (2014). Emotion regulation: quantitative meta-analysis of functional activation and deactivation. Neurosci. Biobehav. Rev. 45, 202–211. doi: 10.1016/j.neubiorev.2014.06.010, PMID: 24984244

[ref17] Gershon A. Kaufmann C. N. Depp C. A. Miller S. Do D. Zeitzer J. M.. (2018). Subjective versus objective evening chronotypes in bipolar disorder. J. Affect. Disord. 1, 342–349. doi: 10.1016/j.jad.2017.08.055 PMC5626649 28843917

[ref18] Gögenur I. Middleton B. Kristiansen V. B. Skene D. J. Rosenberg J. (2007). Disturbances in melatonin and core body temperature circadian rhythms after minimal invasive surgery. Acta Anaesthesiol. Scand. 51, 1099–1106. doi: 10.1111/j.1399-6576.2007.01387.x 17697306

[ref19] Gu Y. F. Bao Z. X. Yu K. H. Wang L. Cheng D. W. Chen S. H.. (2024). Effects of Total intravenous Anesthesia on circadian rhythms in patients undergoing cardiac Transcatheter closure. Zhongguo Yi Xue Ke Xue Yuan Xue Bao 46, 539–545. doi: 10.3881/j.issn.1000-503X.15896, PMID: 38639112

[ref20] Güntürkün O. Ströckens F. Ocklenburg S. (2020). Brain lateralization: a comparative perspective. Physiol. Rev. 100, 1019–1063. doi: 10.1152/physrev.00006.2019, PMID: 32233912

[ref21] Guo R. Ye J. Liao B. Luo X. Rao P. (2023). The relationship between anesthesia and melatonin: a review. Front. Pharmacol. 19:1255752. doi: 10.3389/fphar.2023.1255752 PMC10546185 37795029

[ref22] Hagenauer M. H. Ku J. H. Lee T. M. (2011). Chronotype changes during puberty depend on gonadal hormones in the slow-developing rodent, *Octodon degus*. Horm Behav. 60, 37–45. doi: 10.1016/j.yhbeh.2011.02.004, PMID: 21316365 PMC3112253

[ref23] Harvey J. R. M. Plante A. E. Meredith A. L. (2020). Ion channels controlling circadian rhythms in Suprachiasmatic nucleus excitability. Physiol. Rev. 100, 1415–1454. doi: 10.1152/physrev.00027.2019, PMID: 32163720 PMC7717126

[ref24] Heyde I. Kiehn J. T. Oster H. (2018). Mutual influence of sleep and circadian clocks on physiology and cognition. Free Radic. Biol. Med. 1, 8–16. doi: 10.1016/j.freeradbiomed.2017.11.003 29132973

[ref25] Hodkinson D. J. O’Daly O. Zunszain P. A. Pariante C. M. Lazurenko V. Zelaya F. O.. (2014). Circadian and homeostatic modulation of functional connectivity and regional cerebral blood flow in humans under normal entrained conditions. J. Cereb. Blood Flow Metab. 34, 1493–1499. doi: 10.1038/jcbfm.2014.109, PMID: 24938404 PMC4158665

[ref26] Horne J. A. Ostberg O. (1976). A self-assessment questionnaire to determine morningness-eveningness in human circadian rhythms. Int. J. Chronobiol. 4, 97–110, PMID: 1027738

[ref27] Jones S. E. Lane J. M. Wood A. R. van Hees V. T. Tyrrell J. Beaumont R. N.. (2019). Genome-wide association analyses of chronotype in 697,828 individuals provides insights into circadian rhythms. Nat. Commun. 10:343. doi: 10.1038/s41467-018-08259-7 30696823 PMC6351539

[ref28] Kaufmann C. N. Gershon A. Depp C. A. Miller S. Zeitzer J. M. Ketter T. A. (2018). Daytime midpoint as a digital biomarker for chronotype in bipolar disorder. J. Affect. Disord. 241, 586–591. doi: 10.1016/j.jad.2018.08.032 30172210 PMC6436809

[ref29] Kiernan J. A. Hudson A. J. (1994). Frontal lobe atrophy in motor neuron diseases. Brain 117, 747–757. doi: 10.1093/brain/117.4.747 7922462

[ref30] Kyle S. D. Sexton C. E. Feige B. Luik A. I. Lane J. Saxena R.. (2017). Sleep and cognitive performance: cross-sectional associations in the UK biobank. Sleep Med. 38, 85–91. doi: 10.1016/j.sleep.2017.07.001, PMID: 29031762 PMC5930168

[ref31] Langel J. Ikeno T. Yan L. Nunez A. A. Smale L. (2018). Distributions of GABAergic and glutamatergic neurons in the brains of a diurnal and nocturnal rodent. Brain Res. 1700, 152–159. doi: 10.1016/j.brainres.2018.08.019 30153458

[ref32] Levandovski R. Sasso E. Hidalgo M. P. (2013). Chronotype: a review of the advances, limits and applicability of the main instruments used in the literature to assess human phenotype. Trends Psychiatry Psychother. 35, 3–11. doi: 10.1590/S2237-60892013000100002, PMID: 25923181

[ref33] Li J. Z. Bunney B. G. Meng F. Hagenauer M. H. Walsh D. M. Vawter M. P.. (2013). Circadian patterns of gene expression in the human brain and disruption in major depressive disorder. Proc. Natl. Acad. Sci. USA 110, 9950–9955. doi: 10.1073/pnas.1305814110, PMID: 23671070 PMC3683716

[ref34] Ly J. Q. M. Gaggioni G. Chellappa S. L. Papachilleos S. Brzozowski A. Borsu C.. (2016). Circadian regulation of human cortical excitability. Nat. Commun. 24:11828. doi: 10.1038/ncomms11828 PMC4931032 27339884

[ref35] Meewisse A. J. G. Gribnau A. Thiessen S. E. Stenvers D. J. Hermanides J. van Zuylen M. L. (2024). Effect of time of day on outcomes in elective surgery: a systematic review. Anaesthesia 79, 1325–1334. doi: 10.1111/anae.16395 39108199

[ref36] Meewisse A. J. G. van Huizen E. C. Choi K. F. Kok-de Goede E. N. Turgman O. Schenk J.. (2025). Effects of morning versus afternoon surgery on peri-operative disturbance of sleep-wake timing: An observational study. Acta Anaesthesiol. Scand. 69:e14543. doi: 10.1111/aas.14543, PMID: 39551626

[ref37] Morera-Fumero A. L. Abreu-González P. Henry-Benítez M. Díaz-Mesa E. Yelmo-Cruz S. Gracia-Marco R. (2013). Chronotype as modulator of morning serum melatonin levels. Actas Esp. Psiquiatr. 41, 149–153., PMID: 23803798

[ref38] Niu K. Qin J. L. Lu G. F. Guo J. Williams J. P. An J. X. (2021). Dexmedetomidine reverses postoperative spatial memory deficit by targeting Surf1 and cytochrome c. Neuroscience 1, 148–161. doi: 10.1016/j.neuroscience.2021.04.009 33895343

[ref39] Norbury R. (2021). Diurnal preference and depressive symptomatology: a meta-analysis. Sci. Rep. 11:12003. doi: 10.1038/s41598-021-91205-3 34099766 PMC8184740

[ref40] Orts-Sebastian A. Ludin N. M. Pawley M. D. M. Cheeseman J. F. Warman G. R. (2019). Impact of anaesthesia on circadian rhythms and implications for laboratory experiments. Exp. Neurol. 311, 318–322. doi: 10.1016/j.expneurol.2018.09.017, PMID: 30268768

[ref41] Oster H. Challet E. Ott V. Arvat E. de Kloet E. R. Dijk D. J.. (2017). The functional and clinical significance of the 24-hour rhythm of circulating glucocorticoids. Endocr. Rev. 38, 3–45. doi: 10.1210/er.2015-1080, PMID: 27749086 PMC5563520

[ref42] Poulsen R. C. Warman G. R. Sleigh J. Ludin N. M. Cheeseman J. F. (2018). How does general anaesthesia affect the circadian clock? Sleep Med. Rev. 37, 35–44. doi: 10.1016/j.smrv.2016.12.002, PMID: 28162920

[ref43] Qiu P. Jiang J. Liu Z. Cai Y. Huang T. Wang Y.. (2019). BMAL1 knockout macaque monkeys display reduced sleep and psychiatric disorders. Natl. Sci. Rev. 6, 87–100. doi: 10.1093/nsr/nwz002 34691834 PMC8291534

[ref44] Randler C. Ebenhöh N. Fischer A. Höchel S. Schroff C. Stoll J. C.. (2012). Chronotype but not sleep length is related to salivary testosterone in young adult men. Psychoneuroendocrinology 37, 1740–1744. doi: 10.1016/j.psyneuen.2012.02.008 22425131

[ref45] Roenneberg T. Pilz L. K. Zerbini G. Winnebeck E. C. (2019). Chronotype and social jetlag: a (self-) critical review. Biology (Basel). 8:54. doi: 10.3390/biology8030054 31336976 PMC6784249

[ref46] Roenneberg T. Wirz-Justice A. Merrow M. (2003). Life between clocks: daily temporal patterns of human chronotypes. J. Biol. Rhythm. 18, 80–90. doi: 10.1177/0748730402239679, PMID: 12568247

[ref47] Rosenberg J. Jacobs H. I. L. Maximov I. I. Reske M. Shah N. J. (2018). Chronotype differences in cortical thickness: grey matter reflects when you go to bed. Brain Struct. Funct. 223, 3411–3421. doi: 10.1007/s00429-018-1697-y, PMID: 29948193

[ref48] Rosenberg J. Maximov I. I. Reske M. Grinberg F. Shah N. J. (2014). “Early to bed, early to rise”: diffusion tensor imaging identifies chronotype-specificity. NeuroImage 1, 428–434. doi: 10.1016/j.neuroimage.2013.07.086 24001455

[ref49] Rundshagen I. (2014). Postoperative cognitive dysfunction. Dtsch. Arztebl. Int. 111, 119–125. doi: 10.3238/arztebl.2014.0119, PMID: 24622758 PMC3959222

[ref50] Salehinejad M. A. Wischnewski M. Ghanavati E. Mosayebi-Samani M. Kuo M. F. Nitsche M. A. (2021). Cognitive functions and underlying parameters of human brain physiology are associated with chronotype. Nat. Commun. 12:4672. doi: 10.1038/s41467-021-24885-0 34344864 PMC8333420

[ref51] Scheiermann C. Kunisaki Y. Frenette P. S. (2013). Circadian control of the immune system. Nat. Rev. Immunol. 13, 190–198. doi: 10.1038/nri3386, PMID: 23391992 PMC4090048

[ref52] Schmidt C. Collette F. Cajochen C. Peigneux P. (2007). A time to think: circadian rhythms in human cognition. Cogn. Neuropsychol. 24, 755–789. doi: 10.1080/02643290701754158, PMID: 18066734

[ref53] Smith C. S. Reilly C. Midkiff K. (1989). Evaluation of three circadian rhythm questionnaires with suggestions for an improved measure of morningness. J. Appl. Psychol. 74, 728–738. doi: 10.1037/0021-9010.74.5.728, PMID: 2793773

[ref54] Stangherlin A. (2023). Ion dynamics and the regulation of circadian cellular physiology. Am. J. Physiol. Cell Physiol. 324, C632–C643. doi: 10.1152/ajpcell.00378.2022, PMID: 36689675

[ref55] Taillard J. Sagaspe P. Philip P. Bioulac S. (2021). Sleep timing, chronotype and social jetlag: impact on cognitive abilities and psychiatric disorders. Biochem. Pharmacol. 191:114438. doi: 10.1016/j.bcp.2021.114438, PMID: 33545116

[ref56] Takahashi J. S. Hong H. K. Ko C. H. McDearmon E. L. (2008). The genetics of mammalian circadian order and disorder: implications for physiology and disease. Nat. Rev. Genet. 9, 764–775. doi: 10.1038/nrg2430, PMID: 18802415 PMC3758473

[ref57] Thun E. Bjorvatn B. Osland T. M. Steen V. M. Sivertsen B. Johansen T. A.. (2012). An Actigraphic validation study of seven Morningness-Eveningness inventories. Eur. Psychol. 17, 222–230. doi: 10.1027/1016-9040/a000097

[ref58] Ujma P. P. Scherrer V. (2021). Circadian preference and intelligence – an updated meta-analysis. Chronobiol. Int. 38, 1215–1229. doi: 10.1080/07420528.2021.1926473, PMID: 34015989

[ref59] van Zuylen M. L. Meewisse A. J. G. Ten Hoope W. Eshuis W. J. Hollmann M. W. Preckel B.. (2022). Effects of surgery and general anaesthesia on sleep-wake timing: CLOCKS observational study. Anaesthesia 77, 73–81. doi: 10.1111/anae.15564 34418064 PMC9291940

[ref60] Vink J. M. Groot A. S. Kerkhof G. A. Boomsma D. I. (2001). Genetic analysis of morningness and eveningness. Chronobiol. Int. 18, 809–822. doi: 10.1081/cbi-100107516 11763988

[ref61] Wang J. Li Y. R. Jiang C. Q. Zhang W. S. Zhu T. Zhu F.. (2022). Chronotype and cognitive function: observational study and bidirectional Mendelian randomization. EClinicalMedicine. 1:101713. doi: 10.1016/j.eclinm.2022.101713 PMC9716330 36467458

[ref62] Woodard G. E. Rosado J. A. Li H. (2024). The physiological role of TRP channels in sleep and circadian rhythm. J. Cell. Mol. Med. 28:e18274. doi: 10.1111/jcmm.18274, PMID: 38676362 PMC11053353

[ref63] Xi L. Fang F. Yuan H. Wang D. (2021). Transcutaneous electrical acupoint stimulation for postoperative cognitive dysfunction in geriatric patients with gastrointestinal tumor: a randomized controlled trial. Trials 22:563. doi: 10.1186/s13063-021-05534-9 34425851 PMC8383437

[ref64] Zavada A. Gordijn M. C. Beersma D. G. Daan S. Roenneberg T. (2005). Comparison of the Munich Chronotype questionnaire with the Horne-Ostberg’s Morningness-Eveningness score. Chronobiol. Int. 22, 267–278. doi: 10.1081/cbi-200053536 16021843

[ref65] Zhang J. Zhang Y. Wang J. Xia Y. Zhang J. Chen L. (2024). Recent advances in Alzheimer’s disease: mechanisms, clinical trials and new drug development strategies. Signal Transduct. Target. Ther. 9:211. doi: 10.1038/s41392-024-01911-3 39174535 PMC11344989

